# Do metal implants for total hip arthroplasty affect bioelectrical impedance analysis? A retrospective study

**DOI:** 10.1186/s12891-023-06893-x

**Published:** 2023-09-28

**Authors:** Taku Ukai, Masahiko Watanabe

**Affiliations:** Department of Orthopaedic Surgery, Surgical Science, Tokai University School of Medicine, 143 Shimokasuya, Isehara, 259-1193 Kanagawa Japan

**Keywords:** Bioimpedance, Body composition, Electrical resistance, Total hip arthroplasty

## Abstract

**Background:**

Evaluation of body composition after total hip arthroplasty (THA) is essential because it can be used to evaluate muscle and functional recovery. Muscle volume and degeneration are evaluated using computed tomography (CT). However, CT evaluation of muscle volume has several limitations, such as radiation exposure and high medical cost. Bioelectrical impedance analysis (BIA) has gained attention for resolving these limitations of CT. BIA takes advantage of the microelectric current; thus, metal implants may affect the results. Therefore, this study aimed to elucidate the effects of metal implants on BIA after THA.

**Methods:**

Two groups of patients were assessed (Group 1: 70 patients who underwent unilateral THA and BIA; Group 2: 35 patients who underwent THA and BIA before and after THA). Electric impedance (resistance and reactance) of the operated and non-operated lower limbs was compared in Group 1. The pre- and post-operative impedances of the torso and operated ipsilateral limbs were compared in Group 2.

**Results:**

Regarding electric impedance in Group 1, no significant differences were observed in electrical resistance and reactance between the operated and non-operated lower limbs. Concerning electric impedance in Group 2, postoperative electric resistance of the torso was significantly lower than that preoperatively. However, no significant difference was seen in electric resistance and reactance of the operated ipsilateral limbs preoperatively and postoperatively.

**Conclusions:**

Electrical resistance and reactance of the limbs did not change significantly after THA. BIA is useful for measuring body composition after THA.

## Background

The number of total hip arthroplasties (THAs) has been increasing. THA is one of the most developed surgeries in the orthopedic field and can not only relieve hip pain but also improve gait motion [1]. Muscle strength substantially affects gait, and postoperative muscle evaluation is important in patients after THA. The effectiveness of muscle volume evaluation by using computed tomography (CT) has been previously reported [2–4]. Hounsfield units (HUs) vary depending on the tissue (bone, 400–100 HU; muscle, 30–50 HU; water, 0 HU; fat, -100 HU; air, -1000 HU) [5]. Taking advantage of this feature, physicians have evaluated muscle volume and adipose degeneration using CT [4]. However, CT has several disadvantages such as radiation exposure and increased medical cost.

Bioelectrical impedance analysis (BIA) has attracted considerable attention for resolving these problems associated with CT. Body composition is strongly affected by physical function [1, 6–9] and correlated with osteoarthritis [10], the Barthel index [11], and quadriceps strength [10, 12]. With aging of the population, the number of arthroplasties is also increasing. It is estimated that people have a 7–12% lifetime risk for THA and 8–11% for total knee arthroplasty in the United Kingdom [13]. Therefore, assessment of body composition after arthroplasty will become more important in the near future. Electric impedance consists of resistance and reactance, and BIA devices estimate body composition. Resistance is defined as the power that counteracts electric flow through both direct and alternating currents, and reactance is defined as the power that counteracts the electric flow using only an alternating current. An electric current can easily flow through a fat-free mass containing large amounts of water and electrolytes, but it does not flow through fatty tissue, which contains less water. BIA takes advantage of this principle to estimate the impedance of the body.

BIA enables the measurement of various body conditions such as the fat mass index, free fat mass index, and skeletal muscle volume by applying a harmless electric current to the body. Although BIA can measure body composition easily and rapidly and is more affordable than CT, metal implants such as titanium, steel, and cobalt-chrome can decrease the electrical impedance and affect the results of BIA. However, no studies have elucidated the effects of metal implants on BIA. The primary purpose of this study was to answer the following questions: (1) Is there any difference in electrical impedance between the operated and non-operated limbs? (2) Is there any difference in electrical impedance between the preoperative and postoperative ipsilateral limbs? (3) Is there any difference in electrical impedance between the preoperative and postoperative torso?

## Methods

### Study design and patients

To elucidate the effects of metal implants on BIA, this retrospective study investigated two groups of patients between 2019 and 2023. Group 1 included 70 patients who underwent unilateral THA and BIA postoperatively; Group 2 included 35 patients who underwent unilateral THA and BIA preoperatively and postoperatively. Patients in Group 1 underwent BIA 6 months after THA. Patients in Group 2 underwent BIA 1 month before and 6 months after THA. Patients who had pacemakers or could not remain in the standing position independently were excluded.

### Surgical procedures

#### Total hip arthroplasty

**Fig. 1:**
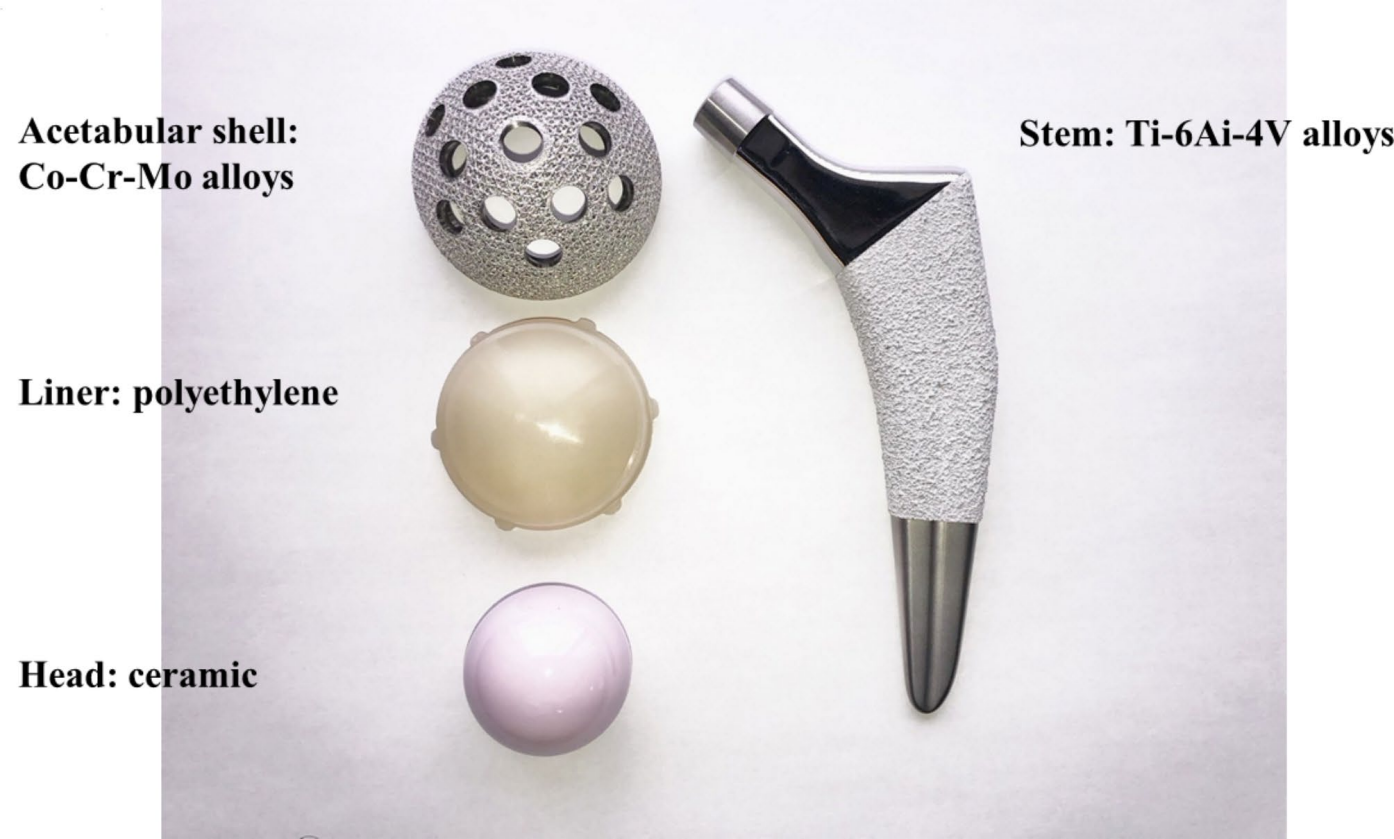
Implant details The implants were divided into four components. The acetabular shell was placed after reaming the acetabulum, comprising a cobalt-chrome alloy. A liner comprising polyethylene without metal was fixed to the cup. The femoral head, composed of ceramic without any metal, was set between the liner and stem. A stem composed primarily of titanium was set after broaching the femur. The components of the metal implants are presented in Table 1

Metal implants (acetabular shell and stem) were placed during THA. The acetabular shells were fixed using two or three screws. Polyethylene liners and ceramic heads were used in all THAs (Fig. 1). The metal compositions of the implants are listed in Table 1.

**Table 1:** Implant composition

Acetabular shell			Stem		
Component	Minimum (%)	Maximum (%)	Component	Minimum (%)	Maximum (%)
Chromium	27	30	Nitrogen		0.05
Molybdenum	5	7	Carbon		0.1
Nickel		0.5	Aluminum	5.5	6.75
Iron		0.75	Iron		0.3
Silicon		1	Vanadium	3.5	4.5
Manganese		1	Titanium		90
Phosphorous		0.02			
Sulfur		0.01			
Nitrogen		0.25			
Aluminum		0.1			
Titanium		0.1			
Boron		0.01			
Cobalt	58	63			

#### Bioelectrical impedance analysis

**Fig. 2:**
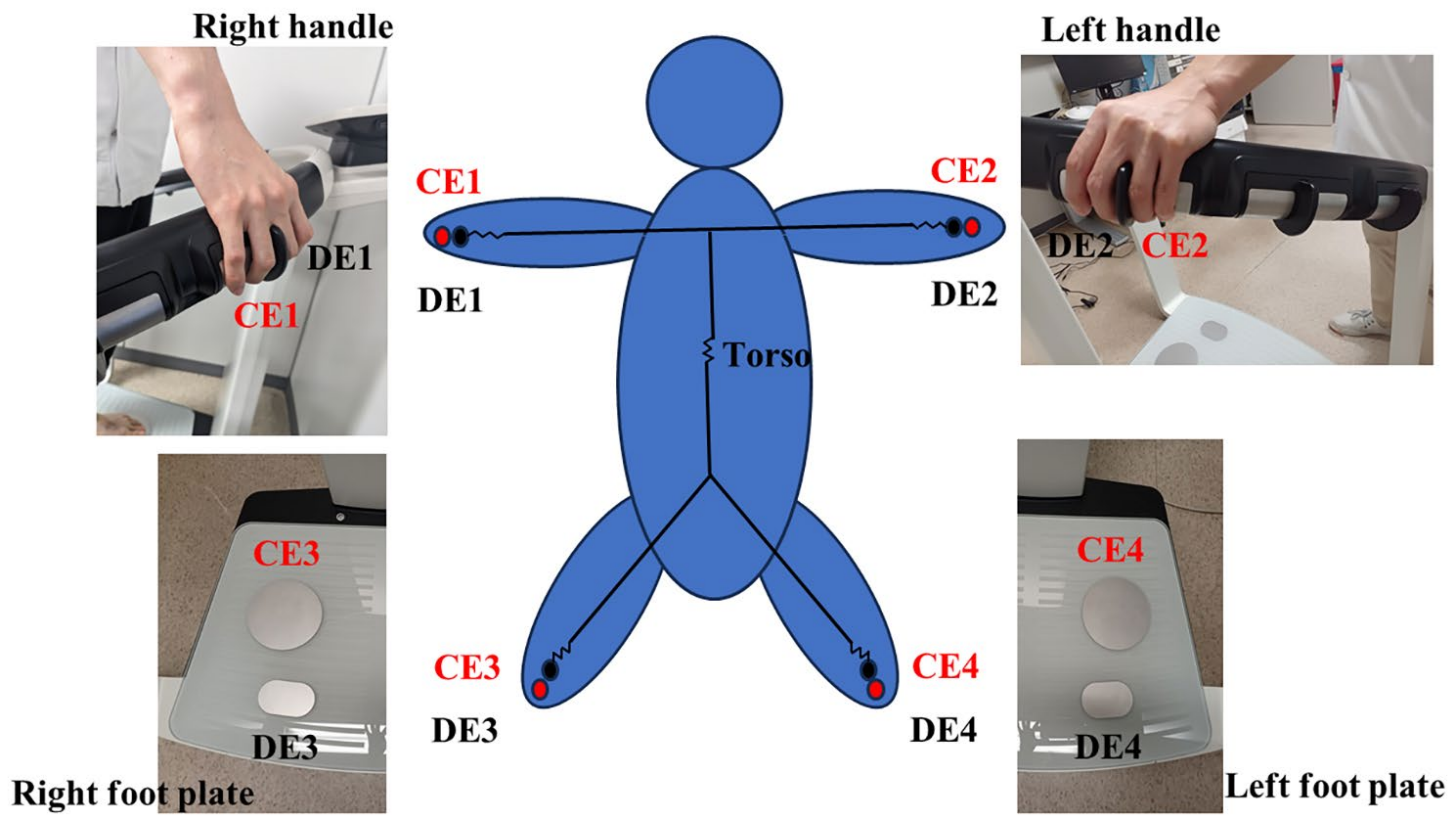
Bioelectrical impedance analysis device The participants stood on two pairs of foot electrodes and gripped the hand electrodes, which emitted a harmless minute of electric current. Electric current flow from a current electrode (CE1, CE2, CE3, and CE4) to a detector electrode (DE1, DE2, DE3, and DE4). Each handle and foot plate has a pair of current and detector electrodes. This device measures the fat mass index, fat-free mass index, and segmental muscle volume (right arm, left arm, trunk, right leg, and left leg). Electricity can readily flow through muscle tissue as its electrical resistance is relatively low due to the presence of water. However, electricity does not flow through adipose tissue due to its high electrical resistance. These properties are helpful for estimating muscle volume

**Fig. 3:**
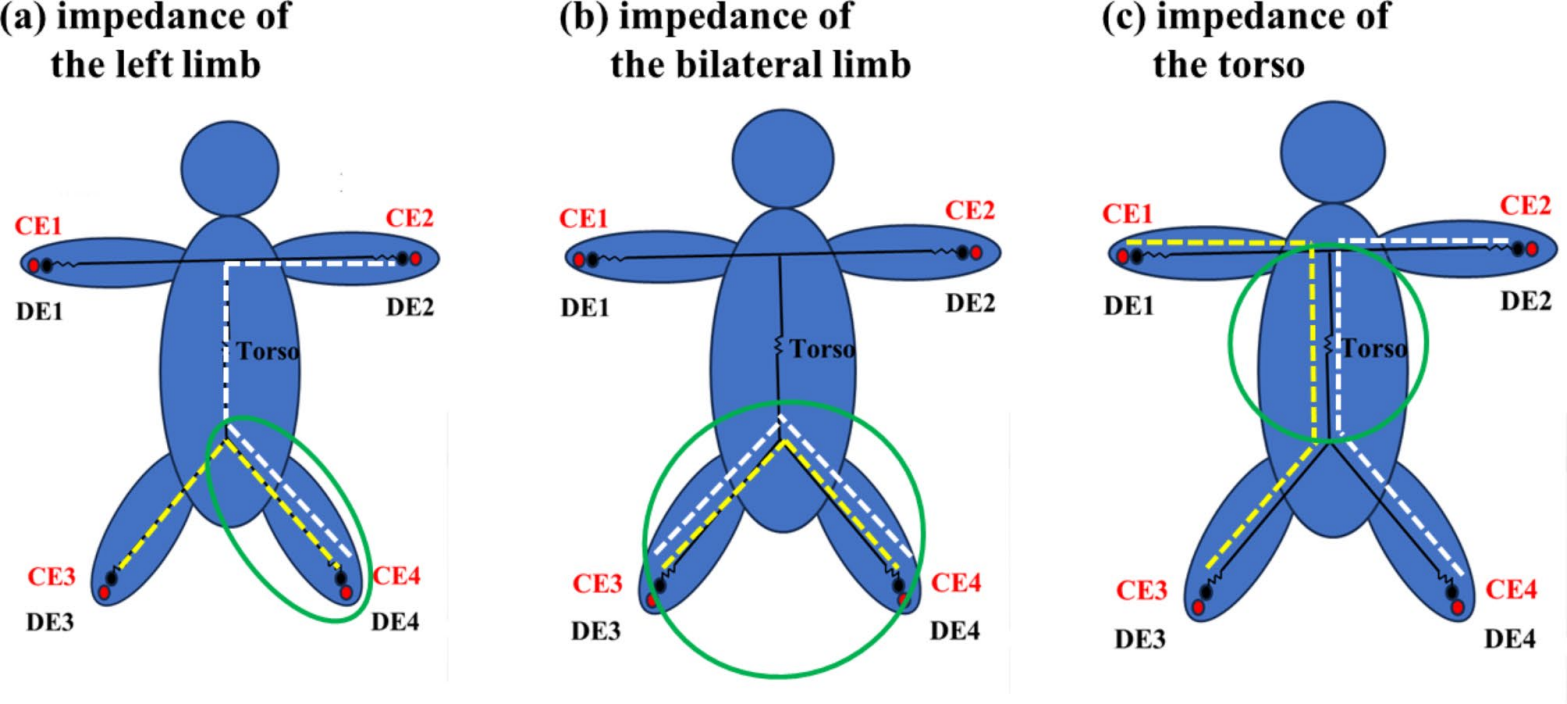
Measurement of body impedance Impedance of each body part was calculated in an overlapping area between the current and voltage. White dotted lines represent the current, whereas yellow dotted lines represent the voltage. For example, when we calculated the impedance of the left leg, current electrodes CE2, CE3, and CE4 and detector electrodes DE2, DE3, and DE4 were used. The overlap area between the current and voltage (green circle) was calculated (**a**). When we calculated the impedance of both limbs, current electrodes CE3 and CE4 and detector electrodes DE3 and DE4 were used, and the overlap area between the current and voltage (green circle) was calculated (**b**). When we calculated the impedance of the torso, all electrodes were used, and the overlap area between the current and voltage (green circle) was calculated (**c**)

BIA was performed using mBCA515 (Seca, Hamburg, Germany). This device was selected as it was constructed for medical application and has been used for evaluation in various research fields such as that for metabolic diseases, cancer, and heart and lung transplantation [14–17]. Moreover, mBCA515 was validated by magnetic resonance imaging [18] and could measure the impedance of each limb and trunk separately at 19 frequencies ranging from 5 to 75 kHz. This device also has eight electrodes, and each handle and footplate has detector and current electrode pairs (Fig. 2). These electrodes were connected to a laptop that could calculate impedance. By constructing a circuit with these electrodes, we could calculate bioelectrical impedance for each body part, including the right arm, right limb, left arm, left limb, both lower limbs, and torso (Fig. 3). The within-day and between-day impedance were measured. Eight participants (four men and four women) underwent BIA three times within the same day to evaluate the within-day impedance variation. They also underwent BIA for three consecutive days to evaluate between-day impedance variation. Participants were instructed to maintain a standing position by attaching all electrodes for 17 s. The bioelectrical resistances of the torso and lower limbs were recorded. BIA was performed under stable temperature and humidity.

#### Statistical analyses

G*Power software (version 3.1.9.2, Germany) was used to calculate the required sample sizes. The parameters of the Mann–Whitney U-test were as follows: two groups; moderate effect size, d = 0.5; alpha error, 0.5; and power, 80%. The required sample size was 106 limbs. The parameters of the Wilcoxon signed-rank test were as follows: one-sample case; moderate effect size, d = 0.5; alpha error, 0.5; and power, 80%. The required sample size was 35.

The Mann–Whitney test was used to compare bioelectrical resistance and reactance between the operated and non-operated lower limbs of patients with THA. The Wilcoxon signed-rank test was used to compare pre- and post-operative bioelectrical resistance and reactance of the torso and ipsilateral lower limbs. All tests were performed at a significance level of *P* < 0.05. Analyses were performed using SPSS statistical software, version 26 (IBM Corp., Armonk, NY, USA).

## Results

Demographic data are presented in Table 2. The lower limb and torso within-day impedance variation was 0.3–1.5% and 0**.7–**2.6% respectively. The lower limb and torso between-day impedance variation was 0.9–2.7% and 0.8**–**2.8**%**, respectively. Regarding the comparison of electrical resistance and reactance between the operated and non-operated lower limbs, slight differences were observed; however, they were not significant. (Table 3). Additionally, in comparing ipsilateral lower limbs preoperatively and postoperatively, slight differences were observed; however, they were not significant (Table 4). In comparing preoperative and postoperative torso, the resistance of the postoperative torso was significantly lower; however, no significant difference was observed between the reactance of the torso preoperatively and postoperatively (Table 5).

**Table 2:** Demographic data

Demographic	Group 1 (*n* = 70)	Group 2 (*n* = 35)
Age (years)	65.9 ± 12.3	66.1 ± 15.1
Sex (male:female)	23:47	11:24
Body mass index (kg/m^2^)	23.8 ± 3.9	23.2 ± 4.3
Diagnosis OA:ION:others	45:19:6	18:14:3

OA, osteoarthritis; ION, idiopathic osteonecrosis of the femoral head

Group 1: Patients who underwent BIA both preoperatively and postoperatively

Group 2: Patients who underwent BIA only postoperatively

All values are expressed as mean ± standard deviation

**Table 3:** Comparison of electrical impedance between the operated and non-operative lower limbs

Resistance (Ω)	Operated	Non-operated	*P*-value
5 kHz	259.7 ± 46.3	258.1 ± 46	0.838
7.5 kHz	257.9 ± 45.9	256.2 ± 45.6	0.841
50 kHz	239.8 ± 42.5	237.5 ± 41.7	0.764
75 kHz	234.8 ± 41.6	232.3 ± 40.8	0.729
**Reactance (Ω)**	**Operated**	**Non-operated**	***P*** **-value**
5 kHz	7.6 ± 2.4	7.8 ± 2.6	0.728
7.5 kHz	9.2 ± 3.0	9.5 ± 3.2	0.683
50 kHz	16.4 ± 4.8	16.9 ± 5.1	0.629
75 kHz	16.3 ± 4.6	16.7 ± 4.8	0.623

All values are expressed as mean ± standard deviation

**Table 4:** Comparison of electrical impedance between the preoperative and postoperative ipsilateral limbs

Resistance (Ω)	Preoperative	Postoperative	*P*-value
5 kHz	253.7 ± 52.2	253.8 ± 39.9	0.829
7.5 kHz	252 ± 51.8	252 ± 39.5	0.784
50 kHz	235.2 ± 47.9	234.3 ± 36.2	0.695
75 kHz	230.5 ± 46.8	229.4 ± 35.3	0.681
**Reactance (Ω)**	**Preoperative**	**Postoperative**	***P*** **-value**
5 kHz	7.0 ± 2.5	7.4 ± 2.6	0.065
7.5 kHz	8.5 ± 3.2	9.0 ± 3.2	0.064
50 kHz	15.5 ± 5.2	16.1 ± 5.0	0.117
75 kHz	15.4 ± 5.0	15.9 ± 4.6	0.150

All values are expressed as mean ± standard deviation

**Table 5:** Comparison of resistance and reactance between the preoperative and postoperative torso

Resistance (Ω)	Preoperative	Postoperative	*P*-value
5 kHz	27.2 ± 3.7	26.3 ± 4.0	0.003
7.5 kHz	27.4 ± 3.7	26 ± 4.0	0.002
50 kHz	24.6 ± 3.4	23.2 ± 3.5	0.002
75 kHz	23.9 ± 3.3	22.6 ± 3.5	0.002
**Reactance (Ω)**	**Preoperative**	**Postoperative**	***P*** **-value**
5 kHz	1.3 ± 0.4	1.3 ± 0.5	0.854
7.5 kHz	1.3 ± 0.4	1.3 ± 0.4	0.829
50 kHz	2.0 ± 0.7	1.9 ± 0.7	0.147
75 kHz	1.9 ± 0.7	1.7 ± 0.7	0.165

All values are expressed as mean ± standard deviation

## Discussion

This study revealed that neither electrical resistance nor reactance of the lower limbs significantly impacted THA. Few reports have evaluated the electrical impedance of metal implants and its effect on BIA measurements [19]. Wagner compared the operated tibial nail side with the non-operated side in a case report [19]. According to the report, the reactance of the operated side decreased by 9.2% and its resistance decreased by 5.2% compared with the non-operated side [19]. Although the electrical resistance of fatty tissue is very high, those of muscle tissue and metal are low. Thus, the postoperative muscle mass may be overestimated after THA. However, only slight difference was observed in electrical resistance and reactance, and no significant difference was observed in this study between the operated and non-operated limbs as well as between the lower limbs preoperatively and postoperatively. Thus, the length of the implant may affect impedance because the resistance of implants varies based on its length. The tibia nail is longer than the stem, and the proportion of the nail implant on the lower limbs is higher than that of arthroplasty. Thus, the implant length and proportion of the implant in the lower limbs may affect impedance. We used a short stem compared with other stems in this study that may have also affected the result; hence, the impedance of a long stem should be elucidated in future research.

Implants are set between the torso and the upper end of the thigh. Although the resistance of the postoperative torso was significantly decreased compared with the preoperative torso, the mean difference value was only 1 ohm; this slight change may be due to alterations in hydration or body composition. One viewpoint is that the torso contains only a part of the screws and does not contain most of the acetabular shell. Another viewpoint is that the postoperative reactance of the torso was not significant. We believe that implants have little effect on bioelectrical impedance. We consider that the resistance and reactance value of the torso was a tenth of that of the lower limbs; thus, only slight change was significant in the torso.

Other studies also performed BIA in patients with fracture [20–22]. Gonzalez-Montalvo et al. performed BIA in patients with hip fractures in the first 72 h after admission and at discharge [21]. However, body composition after a fracture may be quite different from that of degenerative diseases because bleeding directly affects the water balance in the body and the results of BIA. Steihaug et al. evaluated the effect of surgical implants in patients with hip fractures [22]. They compared the electrical impedance between the hospital stay and 3 months after surgery. Their results revealed that although the resistance of the fractured side was significantly decreased in the hospital, there was no significant difference in resistance and reactance between the operated and non-operated limbs at the final follow-up [22]. In addition, resistance and reactance did not vary depending on the surgical implant, such as cannulated screw, hemiarthroplasty, hip compression screw, or THA [22]. This result indicates that BIA is more affected by water balance factors, such as edema, bleeding, and dehydration, than metal implants. However, these studies only performed BIA in patients with hip fractures and only after surgery. We believe that body composition may be affected by fractures and that electrical impedance should be evaluated in non-fractured individuals. Therefore, we included non-fractured individuals and performed postoperative BIA after 6 months to minimize the effect of the operation.

Our report is the first to evaluate the electric impedance preoperatively and postoperatively, and between the operated side and non-operated sides simultaneously. Our results revealed that the resistance and reactance of the limbs did not significantly change after THA. The impedance of the internal body, such as skeletal muscle, cardiac muscle, kidneys, liver, lungs, and spleen, was 171 ohm, 175 ohm, 211 ohm, 342 ohm, 157 ohm, and 405 ohm, respectively [23]. The device used in this study can detect body impedance ranging from 10 to 1000 ohm. However, the impedance of the metal implant was extremely low compared to the detection range of BIA. For example, the impedance of titanium, cobalt, and chrome in THA were 42.7 × 10^− 8^, 6.24 × 10^− 8^, and 12.9 × 10^− 8^ ohm, respectively. Therefore, we consider that BIA cannot detect metal implants and that metal implants do not affect BIA. Additionally, most implants that we used in this study were poor electrical conductors (titanium) and non-conductors (ceramic and polyethylene). Impedance highly varies on the metal type, and the metal length as well as the metal volume may affect the impedance too. Thus, the impedance of the metal type and patients with a metal liner and metal head should be evaluated. The results of this study indicate that BIA is useful for evaluating body composition, even in patients who have undergone THA.

This study had several limitations. First, this study was retrospective, and the sample size was small. To our best knowledge, this is the first study to assess electrical resistance and reactance in patients with THA. Thus, we believe that this study provides new insights into the usefulness of BIA in patients undergoing THA. Second, the metal implants were not unified. However, the composition of the acetabular shell and stem was the same (mainly cobalt, chrome, and titanium), and the electrical resistance and reactance did not vary among implant types [22]. Third, BIA is affected by several factors, such as temperature, exercise, and meals. Therefore, we performed BIA under the same conditions (temperature and humidity) to minimize environmental effects.

## Conclusions

Electrical resistance and reactance did not significantly change between the operated and non-operated sides or between the lower limbs preoperatively and postoperatively. Thus, BIA may be useful for evaluating body composition even after THA.

## Data Availability

The datasets used and/or analyzed during the current study are available from the corresponding author on reasonable request.

## List of Abbreviations

BIA: Bioelectrical impedance analysis
THA: Total hip arthroplasty
CT: Computed tomography
HU: Hounsfield unit
